# Dietary Ferulic Acid Supplementation Improves Antioxidant Capacity and Lipid Metabolism in Weaned Piglets

**DOI:** 10.3390/nu12123811

**Published:** 2020-12-12

**Authors:** Youxia Wang, Xiaoling Chen, Zhiqing Huang, Daiwen Chen, Bing Yu, Jie Yu, Hong Chen, Jun He, Yuheng Luo, Ping Zheng

**Affiliations:** 1 Key Laboratory for Animal Disease-Resistance Nutrition of China Ministry of Education, Institute of Animal Nutrition, Sichuan Agricultural University, Chengdu 611130, China; youxiawang1995@163.com (Y.W.); xlchen@sicau.edu.cn (X.C.); chendwz@sicau.edu.cn (D.C.); ybingtian@163.com (B.Y.); jerryyujie@163.com (J.Y.); hejun8067@163.com (J.H.); luoluo212@126.com (Y.L.); zpind05@163.com (P.Z.); 2 College of Food Science, Sichuan Agricultural University, Yaan 625014, China; chenhong945@sicau.edu.cn

**Keywords:** weaned piglets, ferulic acid, antioxidant capacity, lipid metabolism

## Abstract

Ferulic acid (FA) is a phenolic compound that has antioxidant, hepatoprotective, anticarcinogenic, anti-inflammatory, antiallergic, antimicrobial, antiviral, and vasodilatory effects. This study was conducted to explore the effects of dietary FA supplementation on antioxidant capacity and lipid metabolism in weaned piglets. Eighteen 21-day-old castrated male DLY (Duroc × Landrace × Yorkshire) weaned piglets were randomly divided into control, 0.05%, and 0.45% FA groups. The results showed that, in serum, CAT and T-SOD activities and content of HDL-C were increased, but the content of MDA and the activities of T-CHO and LDL-C were decreased, by FA supplementation. In liver, dietary FA supplementation increased CAT, T-SOD, and GSH-PX activities and upregulated the mRNA levels of *SOD1*, *SOD2*, *CAT*, *GST*, *GPX1*, *GR*, *Nrf2*, *HSL*, *CPT1b*, and *PPARα* but decreased the contents of MDA and TG. Furthermore, dietary FA supplementation increased the protein level of Nrf2, HO-1, and NQO-1. In *longissimus dorsi* muscle, dietary FA supplementation increased the activity of T-SOD and the mRNA abundance of *SOD1*, *SOD2*, *CAT*, *GST*, *GPX1*, *GR,* and *Nrf2* but decreased the contents of MDA and T-CHO. Additionally, dietary FA supplementation increased the protein expressions of Nrf2, HO-1, and NQO1. Together, our data suggest that FA could improve antioxidant capacity and lipid metabolism in weaned piglets.

## 1. Introduction

The content and composition of lipids are in dynamic balance in the body, and the control of lipids by the body is carried out through the lipid metabolism pathway [1]. Lipid metabolism involves a variety of physiological processes, including energy metabolism, transport of lipid-soluble substances, and synthesis of hormones; it is the process of synthesis, decomposition, digestion, absorption, elimination, and transportation of lipids under the action of various enzymes. Lipid metabolism includes the metabolism of fatty acids, triglycerides, and cholesterol. Fatty acid metabolism is the basis of the metabolism of other lipids, and triglyceride and cholesterol metabolism are associated with lipid transporters HDL and LDL [2,3,4]. Liver, adipose tissue, and skeletal muscle are the main organs for fatty acid synthesis and utilization. Normal lipid metabolism plays an important role in maintaining lipid balance in the body. However, in humans, dyslipidemia may increase the risk of cardiovascular disease, obesity, and diabetes [5]. Moreover, the production and elimination of cellular-reactive oxygen species (ROS) are a dynamic balance [6]. When ROS levels rise or antioxidant capacity declines, excessive ROS generation leads to cellular structural damage. ROS contributes to certain chronic conditions, such as cancer [7], diabetes [8], dyslipidemia [9], cardiovascular disease [10], and senescence [11]. However, the body can clear ROS through a powerful antioxidant system and nonenzymatic antioxidants. Many studies have reported that plant extracts such as anthocyanins [12], capsaicin [13], betaine [14], flavonoids [15], and polyphenols [16] improve antioxidant capacity and lipid metabolism of the body, which are negatively associated with cardiovascular risk and have potential benefits in the prevention of cardiovascular disease.

Ferulic acid (FA, 3-methoxy-4-hydroxycinnamic acid) is a phenolic compound that is widely found in the cell walls of vegetables, fruits, and grain plants [17]. Ferulic acid has been reported to have multiple physiological functions, such as antioxidant [18], hepatoprotective [19], anticarcinogenic, anti-inflammatory, antiallergic, antimicrobial, antiviral, vasodilatory, and antithrombotic effects [20,21]. Previous studies have shown that coffee polyphenols and hop polyphenols rich in FA have the effect of inhibiting fat accumulation [22,23]. Dietary supplementation with FA or oryzanol significantly inhibited the weight gain of obese mice induced by high-fat diets and reduced the total weight gain by 47.50% and 26.67%, respectively [24]. In addition, rats fed FA at 30 or 60 mg/kg BW per day showed an improvement in the metabolic syndrome induced by high fat and carbohydrate levels and a decrease in the serum levels of triglyceride (TG) and total cholesterol (TC) [25]. Furthermore, FA is known for its strong antioxidant property, and it has a strong scavenging effect on various oxidized free radicals. FA inhibited lipid peroxidation by peroxy radical or peroxynitrite in a rat brain particle recombination experiment [26]. In vivo studies have shown that supplementation of FA could reduce the content of perhydroxide in the blood and increase the content of GSH and the activities of SOD, CAT, and GPX in the liver, thus improving the antioxidant capacity of diabetic rats [27]. FA has been widely shown to improve the antioxidant properties and lipid metabolism of different rodents, but there has been no report on weaned piglets. Previous studies have shown that there are obvious differences in lipid metabolism characteristics between humans and rodents, and compared to the rodents, pigs have higher homology with humans. Therefore, pigs are the most appropriate animals for studying human nutrition and metabolism [28,29].

Thus, in our present study, we explore the effect of dietary FA supplementation on lipid metabolism and antioxidant capacity and also explain its mechanism.

## 2. Materials and Methods

### 2.1. Ethics Statement

Animal procedures were performed according to the Guidelines for Care and Use of Laboratory Animals of Sichuan Agricultural University and approved by the Animal Care Advisory Committee of Sichuan Agricultural University under permit No. YYS190624.

### 2.2. Animals and Treatment

The 21-day-old castrated male DLY (Duroc × Landrace × Yorkshire) weaned piglets (Sichuan Tieqilishi Industrial Co., Ltd., Mianyang, China) were randomly divided into a control group and 0.05% FA or 0.45% FA supplementation groups according to their body weight, with six piglets in each group. A basal diet was formulated in accordance with NRC (2012) recommendations for the nutritional requirements of 11–25 kg pigs. Ferulic acid (FA, purity ≥99%; Nanjing, China) was obtained from Nanjing Zelang Medical Technology Co. Ltd. All weaned piglets were housed in individual cages (temperature 26–28 °C; humidity 45–75%) and provided with free access to feed and water. The experiment lasted 5 days for adaptation and 5 weeks for the experimental period. The ingredients and composition of the diets are shown in Table A1.

### 2.3. Sample Collection

At the end of the experiment, all weaned piglets fasted for 12 h, and jugular blood was collected. The blood samples were centrifuged at 3000 rpm for 10 min at 4 °C, and then the serum was collected and stored at −20 °C. All piglets were euthanized, and *Longissimus dorsi* (LD) muscle and liver samples were collected, quick-frozen in liquid nitrogen, and stored at −80 °C.

### 2.4. Western Blot

Total protein from LD muscle and liver samples was extracted with RIPA lysis buffer (Beyotime, Shanghai, China). LD muscle and liver were lysed in RIPA lysis buffer for 30 min. After centrifugation at 12,000× *g* for 10 min at 4 °C, the supernatant was collected. A BCA protein assay kit (Pierce, Rockford, IL, USA) and a Nano-Drop ND 2000c spectrophotometer (ThermoFisher Scientific, Waltham, MA, USA) were used to detect the concentration of LD muscle and liver protein. A 5× protein-loading buffer (Beyotime, Shanghai, China) and protein lysates were mixed at 1:4 and denatured in a metal bath at 98 °C for 10 min. Proteins (10 µg) were separated by gel electrophoresis and transferred to a PVDF membrane (Millipore, Eschborn, Germany) using a wet Trans-Blot system (Bio-Rad, Hercules, CA, USA). PVDF membranes were sealed with 5% bovine serum albumin (Beyotime) for 2 h at room temperature and incubated with the following primary antibodies overnight at 4 °C: HO-1 (Proteintech, Cat. No. 66743-1-Ig), Nrf2 (Proteintech, Cat. No. 66504-1-Ig), NQO1 (Proteintech, Cat. No. 67240-1-Ig) or β-actin (Trans, Cat. No. HC201) antibodies. The PVDF membranes were washed with Tris-buffered saline/Tween (TBST) 3 times, for 10 min each time, and incubated with secondary antibody at room temperature for 1.5 h. The protein signals were captured by BeyoECL Plus (Beyotime) and the ChemiDoc XRS imaging system. Protein expressions were calculated by Gel-Pro analyzer and normalized to β-actin protein.

### 2.5. RNA Isolation, cDNA Synthesis, and Real-Time Quantitative PCR

Total RNA from LD muscle and liver samples was extracted with RNAiso Plus reagent (TaKaRa, Dalian, China). RNA concentration was detected by Nano-Drop ND 2000c spectrophotometer (ThermoFisher Scientific, Waltham, MA, USA). Total RNA was reverse-transcribed into cDNA using PrimeScript^®^ RT reagent kit with a gDNA eraser (TaKaRa). Real-time quantitative PCR was performed on a QuantStudio 5 or QuantStudio 6 Flex real-time PCR system (384-cell standard block; Applied Biosystem, Foster, CA, USA), and SYBR Select Master Mix (TaKaRa) was used. Primer sequences used are listed in Table A2. Relative gene expression was evaluated using the 2^−ΔΔCt^ method and normalized to *GAPDH* mRNA.

### 2.6. The Antioxidant Capacity and Malondialdehyde Content Measurement

The activities of total antioxidant capacity (T-AOC), catalase (CAT), glutathione peroxidase (GSH-PX), and total superoxide dismutase (T-SOD) and the level of malondialdehyde (MDA) in LD muscle, liver, and serum were determined by commercial kits (Nanjing Jiancheng Bioengineering Institute, Nanjing, China).

### 2.7. Biochemical Analysis of Serum and Liver Homogenate

The contents of TG, T-CHO, LDL-C, and HDL-C in LD muscle and liver were determined by commercial kits (Nanjing Jiancheng Bioengineering Institute, Nanjing, China).

### 2.8. Statistical Analysis

Data were expressed as mean ± standard error of the mean (SEM) by one-way ANOVA test and Duncan’s multiple-range test (SPSS 25.0, Chicago, IL, USA). A value of *p* < 0.05 represents statistical significance.

## 3. Results

### 3.1. Antioxidant Indices in Serum, LD Muscle, and Liver

We detected antioxidant indicators in serum, LD muscle, and liver. In serum, in comparison with the control group, 0.05% and 0.45% FA supplementation significantly increased CAT activity and reduced the content of MDA. Meantime, 0.45% FA supplementation significantly increased T-SOD activity; however, there was no significant difference between the control group and the 0.05% FA supplementation group (Table 1). In LD muscle, compared with the control group, 0.05% and 0.45% FA supplementation significantly increased T-SOD activity and reduced the content of MDA. However, it was not significant to CAT activity (Table 1). In liver, compared with the control group, 0.45% FA supplementation significantly increased CAT, T-SOD, and GSH-Px activities. However, 0.05% and 0.45% supplementation significantly reduced the content of MDA, and FA supplementation had no effect on T-AOC activity (Table 1).

### 3.2. Expression of Antioxidant Enzyme Gene in LD Muscle and Liver

**Figure 1:**
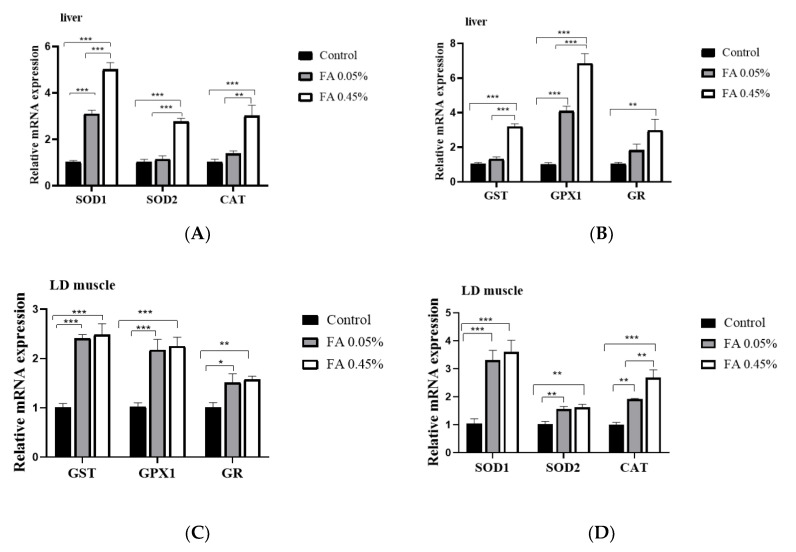
Effect of dietary FA supplementation on the expression of antioxidant enzyme genes in weaned piglets. (**A**,**B**) The mRNA abundance of *SOD1*, *SOD2*, *CAT*, *GST*, *GPX1*, and *GR* in LD muscle. (**C**,**D**) The mRNA abundance of *SOD1*, *SOD2*, *CAT*, *GST*, *GPX1,* and *GR* in liver. The mRNA level of target genes was normalized to the amount of *GAPDH* mRNA. Data were the mean and standard errors (*n* = 6). * *p* < 0.05, ** *p* < 0.01, and *** *p* < 0.001. FA: Ferulic acid, LD: *Longissimus dorsi*, SOD1: superoxide dismutase 1; SOD2: superoxide dismutase 2, CAT: catalase, GST: glutathione S-transferase, GST: glutathione S-transferase, GPX1: glutathione peroxidase 1, GR: glutathione reductase.

Compared with the control group, dietary 0.05% and 0.45% FA supplementation significantly upregulated the mRNA level of SOD1, SOD2, CAT, GST, GPX1, and GR in LD muscle (Figure 1A,B). In addition, in liver, in comparison with the control group, dietary 0.45% FA supplementation significantly increased the mRNA abundance of SOD2, CAT, GST, and GR. Additionally, 0.05% and 0.45% FA supplementation significantly increased the mRNA abundance of SOD1 and GPX1 (Figure 1C,D).

### 3.3. Effect of Ferulic Acid on Nrf2-ARE Signaling Pathways

**Figure 2:**
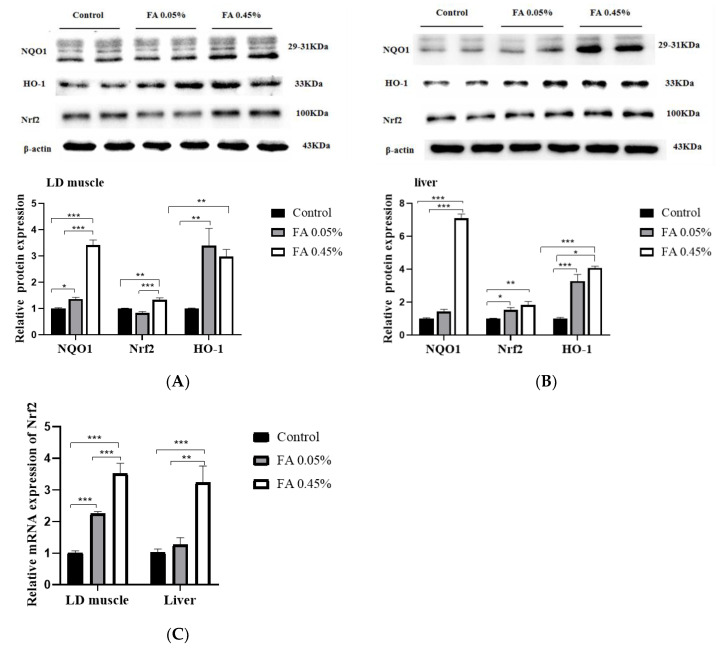
Effect of dietary FA supplementation on the expression of Nrf2-ARE signal components. (**A**,**B**) The protein levels of NQO1, Nrf2, and HO-1 in LD muscle and liver. Equal loading was monitored with an anti-β-actin antibody. (**C**) The mRNA abundance of *Nrf2* in LD muscle and liver. The amount of target gene was normalized to the amount of *GAPDH* mRNA. Data were the mean and standard errors (*n* = 6). * *p* < 0.05, ** *p* < 0.01, and *** *p* < 0.001. NQO1: NAD(P)H, quinone oxidoreductase 1, Nrf2: NF-E2-related nuclear factor 2, HO-1: heme oxygenase-1.

In comparison with the control group, 0.45% FA supplementation significantly (*p* < 0.05) increased the protein levels of Nrf2 and NQO1, and 0.05% and 0.45% FA supplementation significantly upregulated the protein level of HO-1 in LD muscle (Figure 2A). Additionally, compared with the control group, 0.05% and 0.45% FA supplementation significantly increased the protein levels of Nrf2 and HO-1, and 0.45% FA supplementation significantly increased the protein level of NQO1 in liver (Figure 2B). Compared with the control group, 0.05% and 0.45% FA supplementation obviously increased the mRNA abundance of Nrf2 in LD muscle, and 0.45% FA supplementation significantly increased the mRNA abundance of Nrf2 in liver (Figure 2C).

### 3.4. Lipid Profiles of Serum, LD Muscle, and Liver

In serum, compared with the control group, dietary 0.05% and 0.45% FA supplementation obviously decreased the contents of T-CHO and LDL-C and significantly increased the content of HDL-C; however, dietary FA supplementation had no effect on the content of TG (Table 2). In LD muscle, compared with the control group, dietary 0.05% and 0.45% FA supplementation significantly decreased the content of T-CHO but had no difference in the content of TG (Table 2). In liver, in comparison with the control group, dietary 0.45% FA supplementation markedly reduced the content of TG but it did not affect the content of T-CHO (Table 2).

### 3.5. Liver Lipid-Metabolism-Related Gene Expression

**Figure 3:**
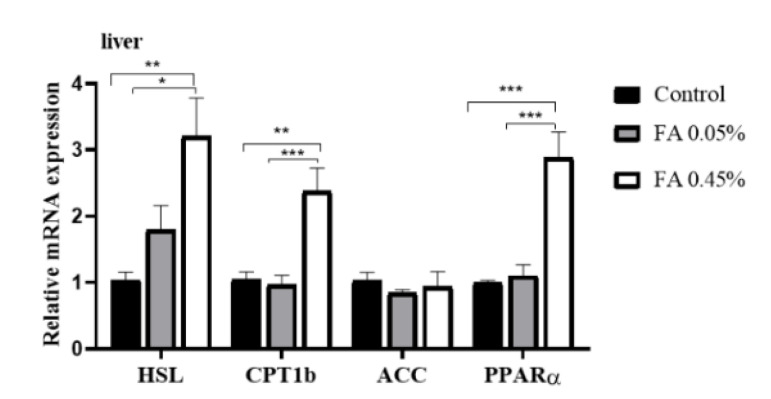
Liver lipid-metabolism-related gene expression. *HSL*, *CPT1b*, *ACC,* and *PPARα* mRNA levels in liver were measured by real-time quantitative PCR and normalized to the amount of *GAPDH* mRNA. Data were the mean and standard errors (*n* = 6). * *p* < 0.05, ** *p* < 0.01, and *** *p* < 0.001.

Compared with the control group, in liver, dietary 0.45% FA supplementation markedly increased the mRNA levels of *HSL*, *CPT1b* and *PPARα*, but FA supplementation did not markedly affect the mRNA abundance of *ACC* (Figure 3).

## 4. Discussion

The disturbance of lipid metabolism causes the dynamic balance of fat deposition and consumption to be disturbed, and it may lead to the ectopic deposition of fat, which may affect the health of animals. In humans, dyslipidemia can lead to fatty deposits in the liver that bring about nonalcoholic fatty liver disease and cardiovascular disease [30,31]. Therefore, maintaining normal lipid metabolism in animals is as important as humans. Meantime, liver, adipose tissue, and skeletal muscle are the main organs for fatty acid synthesis and utilization. Our study suggests that dietary FA supplementation significantly decreased the content of T-CHO in LD muscle, the content of TG in liver, and the content of T-CHO and LDL-C in serum; however, dietary FA supplementation significantly increased the content of HDL-C in serum. Our results are consistent with previous studies on rodents [25]. As we all know, the critical steps of lipid metabolism include lipogenesis, lipolysis, and fatty acid oxidation. Therefore, we further explored the effects of FA supplementation on lipid metabolism in the liver of weaned piglets through these three key steps. Acetyl-CoA carboxylases (ACC) are rate-limiting enzymes in the de-novo synthesis of fatty acids, and they can catalyze acetyl-CoA carboxylation [32]. Neutral lipase hormone-sensitive lipase (HSL) is a rate-limiting enzyme in triacylglycerol (TG) hydrolysis, which can regulate lipolysis [33]. The fatty acids produced by lipid mobilization enter the mitochondria in the form of acylcarnitine, and free carnitine and acyl CoA catalyze are catalyzed by CPT-1b to form long-chain acylcarnitine. In the meantime, the expression of CPT-1b can be regulated by PPARα [34,35]. Our study suggests that dietary FA supplementation has no significant effect on mRNA abundance of *ACC* in liver, which is inconsistent with a previous study, suggesting that the fat-lowering effect of FA might not be mediated by hepatic lipogenesis. However, dietary 0.45% FA supplementation significantly increased the mRNA abundance of *HSL*, *CPT-1b*, and *PPARα* in the livers of weaned piglets, indicating that the fat-lowering effect of FA might be mediated by hepatic lipolysis and fatty acid β-oxidation. Thus, our results suggest that dietary FA supplementation improves the lipid metabolism of weaned piglets.

Chronic oxidative stress is induced by an increase in ROS and a reduction in the activity of antioxidant-related enzymes, and it leads to lipid peroxidation and cardiovascular disease. Hence, it is important to maintain the dynamic balance of ROS clearance and production. The antioxidant system is the central line of defense to protect the body from oxidative stress, and it is activated by multifarious bioactive substances and antioxidant-related genes, including components of the Nrf2-ARE (antioxidant response element) signaling pathway. The activation of the Nrf2-ARE signaling pathway can remove excess ROS from the body [36]. The activated Nrf2-ARE signaling pathway can induce the expression of heme oxygenase-1 (HO-1) [37], NAD(P)H, quinone oxidoreductase 1 (NQO1) [38], superoxide dismutases (SOD) [39], glutathione peroxidases (GSH-Px) [40], and catalases (CAT) [41]. Our present study suggests that dietary 0.45% FA supplementation upregulates the mRNA and protein levels of Nrf2 in weaned piglets. Dietary FA supplementation also increased the protein levels of HO-1 and NQO1. In addition, FA supplementation increased the activity of antioxidant enzymes, such as T-SOD, CAT, and GSH-Px, but decreased the level of MDA. Moreover, the mRNA abundance of *SOD1*, *SOD2*, *CAT*, *GST*, *GPX1*, and *GR* were increased by FA supplementation, so we speculate that dietary FA supplementation might improve the antioxidant capacity of weaned piglets through the Nrf2-ARE signaling pathway. Our results were consistent with a previous study on finishing pigs and rats [24,42].

## 5. Conclusions

In conclusion, our present study indicates that FA could improve lipid profiles and antioxidant capacity in weaned piglets. In addition, we provide evidence that the liver fat-lowering effect of FA might be due to an increase in lipolysis and fatty acid oxidation, and its effect on increasing antioxidant capacity might be attributed to the activation of the Nrf2-ARE signaling pathway.

## Figures and Tables

**Table 1:** Effects of dietary FA supplementation on antioxidant status in serum, LD muscle, and liver of weaned piglets.

Items	Control	FA 0.05%	FA 0.45%
**Serum**			
MDA, nmol/mL	3.02 ± 0.11 ^A^	2.31 ± 0.19 ^B^	2.29 ± 0.11 ^B^
CAT, U/mL	2.64 ± 0.35 ^A^	4.79 ± 0.60 ^B^	4.66 ± 0.43 ^B^
T-SOD, U/mL	297.21 ± 2.33 ^a^	316.03 ± 8.88 ^ab^	334.77 ± 14.35 ^b^
**LD muscle**			
MDA, nmol/mg prot	1.99 ± 0.19 ^A^	1.33 ± 0.07 ^Bb^	0.82 ± 0.12 ^Cc^
CAT, U/mg prot	3.39 ± 0.25	3.52 ± 0.35	3.53 ± 0.12
T-SOD, U/mg prot	30.94 ± 0.93 ^A^	40.33 ± 0.65 ^B^	50.42 ± 2.09 ^C^
**Liver**			
MDA, nmol/mg prot	6.31 ± 0.24 ^A^	4.39 ± 0.17 ^B^	3.10 ± 0.23 ^C^
CAT, U/mg prot	12.38 ± 0.04 ^a^	11.56 ± 0.09 ^Aa^	13.44 ± 0.47 ^Bb^
T-SOD, U/mg prot	837.33 ± 13.90 ^A^	916.59 ± 15.00 ^A^	1121.71 ± 46.57 ^B^
GSH-PX, U/mg prot	66.35 ± 2.13 ^A^	71.14 ± 1.20 ^A^	84.92 ± 4.40 ^B^
T-AOC, U/mg prot	1.30 ± 0.04	1.31 ± 0.11	1.36 ± 0.06

MDA: malondialdehyde; T-AOC: total antioxidant capacity; T-SOD: total superoxide dismutase; GSH-Px: glutathione peroxidase; CAT: catalase, LD: *Longissimus dorsi,* FA: Ferulic acid. Data were the mean and standard errors (*n* = 6). Values within a row with different lowercase letters differ significantly at *p* < 0.05. Values within a row with different capital letters differ significantly at *p* < 0.01.

**Table 2:** Lipid profiles of serum, LD muscle, and liver.

Items	Control	FA 0.05%	FA 0.45%
**Serum**			
TG, mmol/L	0.515 ± 0.062	0.461 ± 0.052	0.504 ± 0.027
T-CHO, mmol/L	3.865 ± 0.347 ^Aa^	2.806 ± 0.236 ^b^	2.703 ± 0.215 ^B^
LDL-C, mmol/L	1.819 ± 0.165 ^A^	1.288 ± 0.069 ^B^	1.320 ± 0.070 ^B^
HDL-C, mmol/L	2.623 ± 0.106 ^A^	4.293 ± 0.171 ^B^	4.031 ± 0.123 ^B^
**LD muscle**			
TG, mmol/mg prot	0.121 ± 0.010	0.146 ± 0.026	0.198 ± 0.041
T-CHO, mmol/mg prot	0.132 ± 0.017 ^A^	0.040 ± 0.007 ^B^	0.046 ± 0.004 ^B^
**Liver**			
TG, mmol/mg prot	0.149 ± 0.007 ^a^	0.147 ± 0.006 ^a^	0.128 ± 0.003 ^b^
T-CHO, mmol/mg prot	0.051 ± 0.006	0.047 ± 0.005	0.045 ± 0.005

T-CHO: total cholesterol; TG: triglyceride; LDL-C: Low-density lipoprotein–cholesterol; HDL-C: high-density lipoprotein–cholesterol. Data were the mean and standard errors (*n* = 6). Values within a row with different lowercase letters differ significantly at *p* < 0.05. Values within a row with different capital letters differ significantly at *p* < 0.01.

## Appendix A

**Table A1:** Composition and nutrient levels of the basal diet.

Ingredients	Content	Nutrient Levels	Content
Corn (%)	29.70	Digestible energy (Mcal/kg)	3.54
Extruded corn (%)	30.00	Crude protein (%)	20.0
Soybean oil (%)	1.39	Calcium (%)	0.75
Sucrose (%)	2.50	Available P (%)	0.37
Whey power (%)	5.00	Digestible Lys (%)	1.31
Dehulled soybean meal (%)	7.50	Digestible Met (%)	0.39
Extruded soybean (%)	10.00	Digestible Thr (%)	0.78
Soybean protein concentrate (%)	6.46	Digestible Trp (%)	0.21
Fish meal (%)	5.00		
L-Lysine·HCl (%)	0.43		
DL-Methionine (%)	0.09		
L-Threonine (%)	0.16		
L-Tryptophan (%)	0.02		
Choline chloride (%)	0.10		
Calcium powder (%)	0.66		
Dicalcium phosphate (%)	0.34		
NaCl (%)	0.30		
Vitamin premix ^a^ (%)	0.05		
Mineral premix ^b^ (%)	0.30		
Total	100.00		

^a^ The vitamin premix provides per kg of diet: VA 8000 IU; VD3 2000 IU; VE 20 IU; VB1 1.5 mg; VB2 5.6 mg; VB12 0.02 mg; VB6 1.5 mg; calcium pantotenate 10 mg; nicotinic acid 15 mg; biotin 0.1 mg; folic acid 0.6 mg. ^b^ The mineral premix provides per kg of diet: Cu (CuSO_4_.5H_2_O) 6 mg; Fe (FeSO_4_.H_2_O) 100 mg; Zn (ZnSO_4_.7H_2_O) 100 mg; Mn (MnSO_4_) 4 mg; I (KI) 0.14 mg; Se (Na_2_SeO_3_) 0.3 mg.

**Table A2:** Primer sequences used for real-time quantitative PCR.

Genes	Primer	Sequence (5′-3′)	GenBank Accession No.	Size (bp)
SOD1	Forward Reverse	AGACCTGGGCAATGTGACTG GTGCGGCCAATGATGGAATG	NM_001190422	102
SOD2	Forward Reverse	TGTAACTGAGCGATACGCCG GGTATTCGGCGCTCCTACAA	NM_214127	99
GPX1	Forward Reverse	GTGAATGGCGCAAATGCTCA ATTGCGACACACTGGAGACC	NM_214201	126
GST	Forward Reverse	CCAACCCAGAAGACTGCTCA CATTCAGGTGGGCTCTTCGT	AB000884	102
Nrf2	Forward Reverse	GGAGCTGTTGATCTGTTGCG TCCATGTCCCTTGACAGCAA	XM_003133500	132
GR CAT	Forward Reverse Forward Reverse	GTGAGCCGACTGAACACCAT CAGGATGTGAGGAGCTGTGT CAGATGAAGCATTGGAAGGAGC TTGTCTCCTATCGGATTCCCAG	AY368271 NM_214301	141 83
ACC	Forward Reverse	ACCGAATTGGTTCCTTTGGAC CCAGTCCGATTCTTGCTCCA	AF175308	123
HSL	Forward Reverse	CCCATCCTCTCCATCGACT CAGCAGTAGGCGTAGAAGCAC	NM_214315	83
CPT1b	Forward Reverse	TGACTCGAATGTTCCGGGAG AGATCTTGCAGGTCTGCTTTCA	NM_001007191	118
PPARα	Forward Reverse	GAGTTCGCCAAGTCCATCC CCGTCCTTGTTCATCACAGAG	NM_001044526	122
GAPDH	Forward Reverse	ACACTGAGGACCAGGTTGTG GACGAAGTGGTCGTTGAGGG	NM_001206359	98

GAPDH: glyceraldehyde phosphate dehydrogenase; ACC: acetyl-CoA carboxylase; HSL: hormone sensitive lipase; PPARα: peroxisome proliferator-activated receptor α; CPT1b: carnitine palmitoyltransferase-1b; SOD1: superoxide dismutase 1; SOD2: superoxide dismutase 2; CAT: catalase; GPX1: glutathione peroxidase 1; GST: glutathione S-transferase; Nrf2: NF-E2-related nuclear factor 2; GR: glutathione reductase.
